# Supplementary material for: A positive feedback loop between Myc and aerobic glycolysis sustains tumor growth in a Drosophila tumor model
Source: eLife. 2019 Jul 1;8:e46315. doi: 10.7554/eLife.46315 (PMC6636907; doi:10.7554/eLife.46315)
Supplement: Supplementary file 1. [file elife-46315-supp1.docx]

**Supplementary File 1. A list of *Drosophila* genes studied in this work with their human homologs.**

| *Drosophila* genes | Fly Annotation symbol | Human homolog(s) | Description |
| --- | --- | --- | --- |
| **Glycolysis** | | | |
| *glut1* | CG43946 | *SLC2A3, SLC2A1, SLC2A4, SLC2A14, SLC2A2* | Glucose transporter |
| *hex-a* | CG3001 | *GCK, HK1, HK2, HK3, HKDC1* | Hexokinase |
| *hex-c* | CG8094 | *GCK, HK1, HK2, HK3, HKDC1* | Hexokinase |
| *pgi* | CG8251 | *GPI* | Glucose-6-phosphate isomerase |
| *pfk* | CG4001 | *PFKM, PFKP, PFKL* | Phosphofructokinase |
| *pfk2*  (or *pfrx*) | CG3400 | *PFKFB3, PFKFB1, PFKFB2, PFKFB4* | 6-Phosphofructo-2-kinase/fructose-2,6-bisphosphatase |
| *ald*  (or *ald1*) | CG6058 | *ALDOA, ALDOC, ALDOB* | Aldolase |
| *tpi* | CG2171 | *TPI1* | Triosephosphate isomerase |
| *gapdh1* | CG12055 | *GAPDH, GAPDHS* | Glyceraldehyde-3-phosphate dehydrogenase |
| *gapdh2* | CG8893 | *GAPDH, GAPDHS* | Glyceraldehyde-3-phosphate dehydrogenase |
| *pgk* | CG3127 | *PGK1, PGK2* | Phosphoglycerate kinase |
| *pglym78* | CG1721 | *PGAM1, PGAM2, PGAM4* | Phosphoglycerate mutase |
| *eno* | CG17654 | *ENO1, ENO2, ENO3* | Enolase |
| *pyk* | CG7070 | *PKM, PKLR* | Pyruvate kinase |
| *Ldh*  (or *impl3*) | CG10160 | *LDHA, LDHB, LDHAL6A, LDHC, LDHAL6B* | Lactate dehydrogenase |
| **Cell signaling** | | | |
| *dMyc* | CG10798 | *MYC, MYCL, MYCN* | Transcription factor, cell growth, cell competition and regenerative proliferation |
| *sima* | CG45051 | *HIF1A, HIF2A, HIF3A* | Hypoxia-inducible factors |
| *yki* | CG4005 | *YAP1, WWTR1 (TAZ)* | Effectors of the Hippo tumor suppressor pathway |
| *arm* | CG11579 | *CTNNB1 (β-catenin), JUP (γ-catenin)* | Cell adhesion and Wingless signaling |
| *ci* | CG2125 | *GLI3, GLI2, GLI1* | Effectors of Hedgehog signaling |
| *N* | CG3936 | *NOTCH1, NOTCH2, NOTCH3, NOTCH4* | Notch receptors in Notch signaling |
| *ex* | CG4114 | *FRMD1, FRMD6* | A FERM-domain containing protein, target gene and positive regulator of the Hippo signaling |
